# Correlation Between Atopic Dermatitis and Sleep Quality Among Adults in Saudi Arabia

**DOI:** 10.7759/cureus.12051

**Published:** 2020-12-13

**Authors:** Waad Alomayri, Nawal Alanazi, Fatma Faraj

**Affiliations:** 1 Department of Family Medicine, Family Medicine Residency Training Program, King Salman Armed Forces Hospital, Tabuk, SAU; 2 Department of Family Medicine, King Salman Armed Forces Hospital, Tabuk, SAU

**Keywords:** quality of sleep, atopic dermatitis, quality of life, saudi arabia

## Abstract

Background

Atopic dermatitis is a chronic inflammatory skin disease characterized by pruritic, dry, and eczematous lesions. The effect of atopic dermatitis on the quality of patients’ lives has been investigated. However, its impact on the quality of sleep is still controversial.

Objective

This study aims to identify the correlation between sleep quality and atopic dermatitis disease among adults living in Saudi Arabia.

Design and setting

This is a cross-sectional, quantitative survey study, carried out during July and August 2020, which included atopic dermatitis patients. The study used Arabic versions of a self-administered, Internet-based questionnaire of the Pittsburgh Sleep Quality Index (PSQI) and Dermatology Life Quality Index (DLQI). Face-to-face patient interviews were not applicable due to the COVID-19 pandemic.

Results

A total of 400 patients participated in this survey study. Eighty-six percent (86%) were females while 39.6% were in the age group of 18 to 25 years old. A third of the responders could not sleep within half an hour, and a third of them woke up at night or early in the morning more than three times a week. Twenty-three point eight percent (23.8%) of the responders rated their sleep quality as a very good quality of sleep while 17.8% rated it as very bad. Inability to get to sleep within half an hour (p-value=0.002), waking up at night (p-value=0.005), and not being able to sleep because of pain (p-value<0.001) were all significantly correlated to the occurrence of many or a lot of skin symptoms of atopic dermatitis. There was a significantly higher total score among patients with atopic dermatitis (p value<0.001), which shows a poorer quality of sleep.

Conclusion

Symptoms of atopic dermatitis negatively influence the quality of sleep of adults in Saudi Arabia. The frequency of symptoms are significantly correlated to the poor quality of sleep. Therefore, we suggest that the evaluation of sleep quality is necessary for the management of atopic dermatitis patients.

## Introduction

Atopic dermatitis (AD) is a chronic inflammatory skin disease characterized by pruritic, dry, and eczematous lesions [1]. The prevalence of adult AD ranged from 2.1% to 4.9% across all countries. Prevalence is generally higher in females relative to males and is lower among older age groups, with a peak prevalence most frequently observed in the 25 to 34-year and 35 to 44-year age groups [2]. AD affects all races and geographic locations [3]. Their pathogenesis, however distinct, is multifactorial and encompasses a hyperactive immune system, environmental factors, and genetic predisposition [4]. Regarding the genetic predisposition, many patients affected by AD have a family history of atopies such as asthma, food allergies, AD, or hay fever [3].

Pruritus is an essential symptom of AD, so much so that AD is known as “the itch that rashes,” which can affect sleep, quality of life (QoL), and different aspects of well-being in patients with AD. Current published data demonstrate that 33%-87.1% of adults with AD suffer from sleep disturbance based on surveys of AD patients. Sleep insufficiency can lead to excessive daytime sleepiness, mood disturbance, and impaired cognition, which can then have an impact on work or school productivity, accidents, and adverse health outcomes, including metabolic, endocrine, and immune dysregulation, such as type 2 diabetes, high blood pressure and infection [5].

## Materials and methods

Study design

A cross-sectional study design was applied during July and August 2020.

Study population

Atopic dermatitis patients living in Saudi Arabia at the time of study conduction were eligible for inclusion in the study.

Sample size 

We have used the following equation to calculate the sample size: 

n = z^2^*p (1-p) / ε​​​​​​^2^

z is the z score ε is the margin of error N is the population size p̂ is the population proportion

The calculated minimum sample size is:

 n = (1.96)^2^*0.5 (1-0.5) / (0.05)^2 ^= 384.16

A sample size of at least 385 people would be necessary.

Data collection tool 

The study used the Arabic forms of a self-administered, Internet-based questionnaire of the Pittsburgh Sleep Quality Index (PSQI) and Dermatology Life Quality Index (DLQI). All patients provided electronic informed consent; permission to use the questionnaires was obtained through an e-mail communication with the authors.

Patients with AD were identified by the self-reported presence of AD by answering “yes” to the question “Have you been diagnosed with atopic dermatitis.” This question, which required an affirmative answer, ensured the sample provided a truly representative population of patients with self-reported AD.

The PSQI questionnaire includes the demographic data of the respondents, subjective sleep quality, sleep latency, sleep duration, habitual sleep efficiency, sleep disturbances, use of sleeping medication, and daytime dysfunction. A global score of ≥ 5 reflects a specific and sensitive measure of poor sleep quality [4].

The DLQI includes 10 questions, covering the following topics: symptoms, embarrassment, shopping and home care, clothes, social and leisure, sport, work or study, close relationships, sex, and treatment. Each question refers to the impact of the skin disease on the patient’s life over the last week.

Data entry and analysis

All collected data were entered and analyzed using the Statistical Package for Social Science (SPSS) version 26. A p-value of less than or equal to 0.05 was utilized as a cut-off for statistical significance. A necessary statistical test was used as appropriate, e.g., the chi-squared test.

## Results

This quantitative study included 400 patients who had been diagnosed with atopic dermatitis in Saudi Arabia. The self-administered questionnaire was carried out between July and August 2020. All descriptive and comparative analyses are shown below.

Demographics of patients

**Figure 1:**
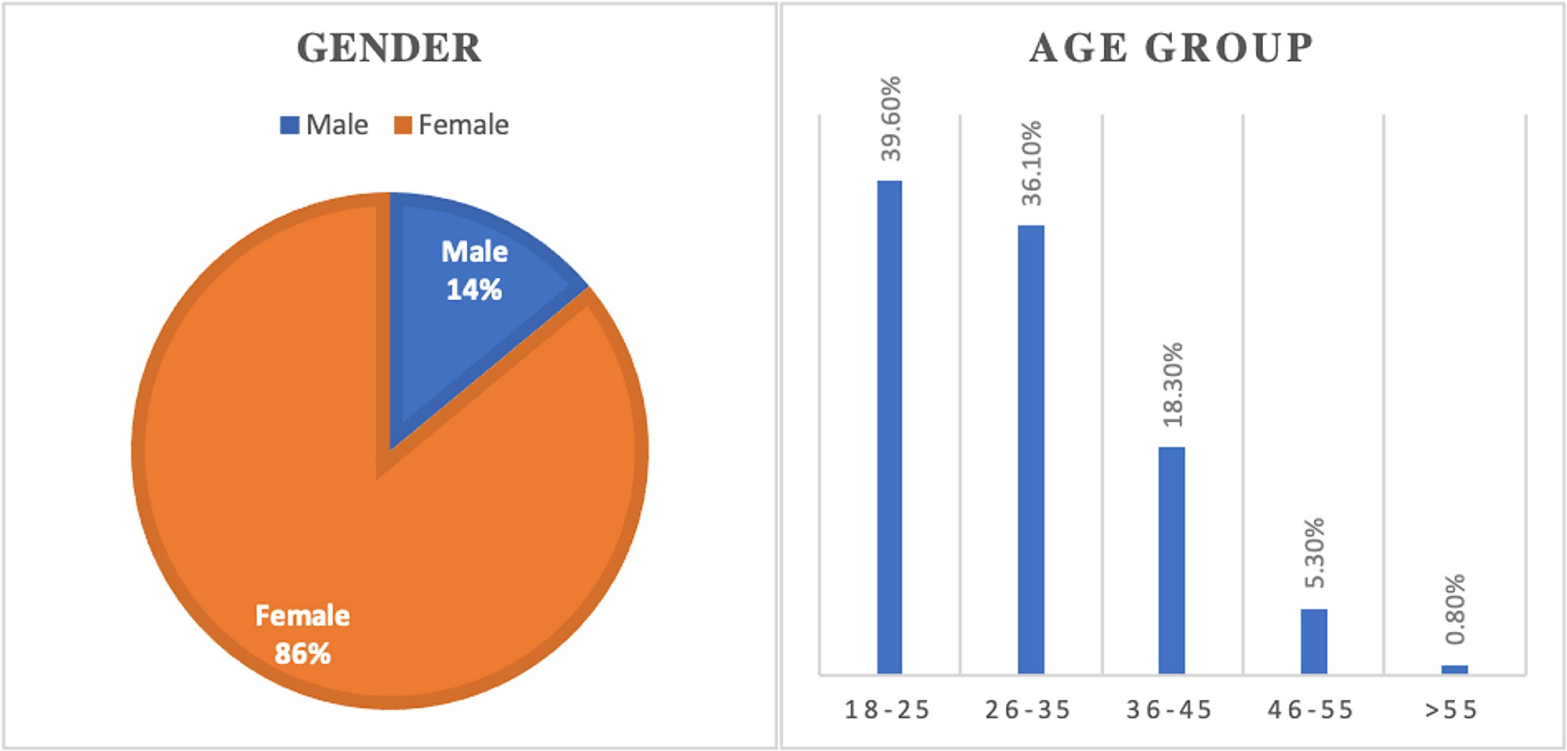
Demographics of patients.

Among the 400 patients, 86% were females while age was subcategorized into five age groups. Thirty-nine point six percent (39.60%) of the responders were in the age group of 18 to 25 years old, as shown in Figure 1.

Sleep disturbances during the last month

Participants were asked about their sleep habits and routines during the last month. Almost a third of the responders could not fall asleep within half an hour. Additionally, about a third of the responders woke up at night or early in the morning more than three times a week. Additionally, 16% of the responders could not sleep because of pain, 6% used over-the-counter medications (OTC) or prescribed medicine to sleep, and 6.5% had difficulty staying awake during different activities three or more times a week, as shown in Table 1.

**Table 1:** Sleep disturbances during the last month OTC: over the counter

Item description		Count	Percent
Could not sleep within 30 minutes	Not during the last month	74	18.5
	Less than once a week	63	15.8
	Once or twice a week	110	27.6
	Three times or more/week	153	38.1
Waking up in the middle of the night or early in the morning	Not during the last month	84	21.1
	Less than once a week	82	20.6
	Once or twice a week	112	28.1
	Three times or more/week	122	30.3
Waking up to go to the bathroom	Not during the last month	94	23.6
	Less than once a week	116	28.8
	Once or twice a week	86	21.6
	Three times or more/week	104	26.1
Cannot breathe comfortably	Not during the last month	228	56.9
	Less than once a week	57	14.3
	Once or twice a week	70	17.5
	Three times or more/week	45	11.3
Coughing or loud snoring	Not during the last month	309	77.2
	Less than once a week	57	14.3
	Once or twice a week	16	4.0
	Three times or more/week	18	4.5
Feeling very cold	Not during the last month	169	42.1
	Less than once a week	112	28.1
	Once or twice a week	72	18.0
	Three times or more/week	47	11.8
Feeling very hot	Not during the last month	133	33.1
	Less than once a week	93	23.3
	Once or twice a week	105	26.3
	Three times or more/week	69	17.3
Have bad dreams or nightmares	Not during the last month	145	36.1
	Less than once a week	110	27.6
	Once or twice a week	86	21.6
	Three times or more/week	59	14.8
Have pain	Not during the last month	156	38.8
	Less than once a week	102	25.6
	Once or twice a week	78	19.5
	Three times or more/week	64	16.0
Need prescribed or OTC medications to sleep	Not during the last month	320	79.9
	Less than once a week	39	9.8
	Once or twice a week	17	4.3
	Three times or more/week	24	6.0
Difficulty staying awake while driving, eating meals, or engaging in social activities	Not during the last month	221	55.1
	Less than once a week	88	22.1
	Once or twice a week	65	16.3
	Three times or more/week	26	6.5
Had a problem to keep up enough enthusiasm	Three or more times a week	39	9.8
	Once or twice a week	100	25.1
	Less than once a week	150	37.3
	Not during the past month	111	27.8

Participants were also asked to rate their sleep during the last month; 23.8% of the responders had a very good quality of sleep while 17.8% had a very bad quality of sleep, as shown in Table 2.

**Table 2:** Evaluation of AD patients for their quality of sleep AD: atopic dermatitis

Item description		Count	Percent
Overall sleep quality	Very good	95	23.8
	Fairly good	178	44.4
	Fairly bad	56	14.0
	Very bad	71	17.8

Furthermore, it has been shown that the most common time of going to sleep among the included cohort was 1:00 AM, where the average time taken to fall asleep each night was 23.4±10.8 minutes. While the most common time of waking up was 8:00 AM, and the mean number of hours of actual sleep was 7.3±2.6 hours. Also, the mean number of times of interrupted sleep for causes outside of AD was 3.2±1.1 times.

Impact of atopic dermatitis on the quality of life

Responders were also asked about how their atopic dermatitis symptoms are affecting their daily life activities. Almost a third of the patients described that their skin symptoms cause them some amount of pain or soreness, shame or embarrassment, or interference with social, sports, and other activities.

Additionally, only 10.8% of the patients found that their skin condition had affected their study or work, where 6.4% of these patients described the impaction as severe and significant.

Furthermore, less than a third of the patients described that their symptoms caused them little problems with their family and friends, sexual problems, or at home.

Correlation between the presence of eczema and quality of sleep

Patients' responses to questions related to the quality of sleep were compared over the presence of atopic dermatitis symptoms for the included patients using chi-square testing at the level of significance p-value <0.05.

It has been shown that an inability to get to sleep within half an hour (p-value=0.002), waking up at night (p-value=0.005), and not being able to sleep because of pain (p-value<0.001) were all significantly correlated to the occurrence of many or a lot of skin symptoms of atopic dermatitis, as shown in Table 3.

**Table 3:** Correlation between atopic dermatitis (AD) symptoms frequency in the Dermatology Life Quality Index (DLQI) and each question in Pittsburgh Sleep Quality Index (PSQI)

PSQI items		Itchy, painful, sore, or tingling					p-value
		Very much	A lot	A little		Not at all	
Could not sleep within 30 minutes	Less than once a week	16.9%	18.5%		11.4%	10.5%	0.002*
	Once or twice a week	36.4%	28.7%		17.1%	21.1%	
	3 or more times a week	53.3%	33.8%		33.2%	26.3%	
	Not during the last month	14.4%	19.1%		18.1%	42.1%	
Waking up in the middle of the night or early in the morning	Less than once a week	23.7%	22.3%		12.4%	31.6%	0.005*
	Once or twice a week	33.1%	26.8%		27.6%	10.5%	
	3 or more times a week	44.8%	24.2%		25.4%	31.6%	
	Not during the last month	17.8%	26.8%		15.2%	26.3%	
Waking up to go to the bathroom	Less than once a week	23.8%	35.6%		28.0%	21.1%	0.043*
	Once or twice a week	21.0%	16.1%		25.5%	26.3%	
	3 or more times a week	36.2%	27.1%		19.7%	15.8%	
	Not during the last month	19.0%	21.2%		26.8%	36.8%	
Cannot breathe comfortably	Less than once a week	9.3%	22.9%		19.0%	15.8%	0.018*
	Once or twice a week	12.7%	12.1%		20.0%	10.5%	
	3 or more times a week	14.4%	15.2%		6.4%	10.5%	
	Not during the last month	63.6%	58.6%		45.7%	63.2%	
Coughing or loud snoring	Less than once a week	14.4%	11.5%		20.0%	5.3%	0.011*
	Once or twice a week	8.5%	3.2%		1.0%	0.0%	
	3 or more times a week	8.6%	3.2%		3.46%	0.0%	
	Not during the last month	73.7%	82.2%		70.5%	94.7%	
Feeling very cold	Less than once a week	28.8%	26.1%		26.7%	47.4%	0.037*
	Once or twice a week	18.6%	17.2%		21.0%	5.3%	
	3 or more times a week	12.7%	19.0%		7.0%	5.3%	
	Not during the last month	39.8%	49.7%		33.3%	42.1%	
Feeling very hot	Less than once a week	25.4%	25.5%		16.2%	31.6%	<0.001*
	Once or twice a week	28.0%	26.1%		25.7%	21.1%	
	3 or more times a week	14.4%	33.3%		10.2%	5.3%	
	Not during the last month	32.2%	38.2%		24.8%	42.1%	
Have bad dreams or nightmares	Less than once a week	25.7%	22.9%		32.5%	26.3%	0.440
	Once or twice a week	22.9%	25.4%		17.8%	21.1%	
	3 or more times a week	19.0%	16.9%		10.8%	10.5%	
	Not during the last month	32.4%	34.7%		38.9%	42.1%	
Have pain	Less than once a week	31.4%	23.6%		23.8%	15.8%	<0.001*
	Once or twice a week	20.3%	16.6%		23.8%	15.8%	
	3 or more times a week	16.1%	28.6%		8.9%	5.3%	
	Not during the last month	32.2%	51.0%		23.8%	63.2%	
Need over-the-counter medicines to sleep	Less than once a week	11.4%	10.2%		7.6%	15.8%	0.530
	Once or twice a week	2.9%	5.1%		5.1%	0.0%	
	3 or more times a week	8.6%	7.6%		3.8%	0.0%	
	Not during the last month	77.1%	77.1%		83.4%	84.2%	
Difficulty staying awake while driving, eating meals, or engaging in social activities	Less than once a week	27.6%	19.5%		21.0%	15.8%	0.072
	Once or twice a week	16.2%	22.0%		12.7%	10.5%	
	3 or more times a week	10.5%	6.8%		4.5%	0.0%	
	Not during the last month	45.7%	51.7%		61.8%	73.7%	
Had a problem to keep up enough enthusiasm	Less than once a week	36.2%	42.4%		34.4%	36.8%	0.093
	Once or twice a week	28.6%	29.7%		20.4%	15.8%	
	3 or more times a week	11.4%	3.4%		13.4%	10.5%	
	Not during the last month	23.8%	24.6%		31.8%	36.8%	
Overall sleep quality	Very good	16.2%	23.7%		28.7%	26.3%	0.507
	Good	43.8%	45.8%		43.9%	42.1%	
	Bad	16.2%	12.7%		13.4%	15.8%	
	Very bad	23.8%	17.8%		14.0%	15.8%	

Moreover, the PSQI global score was also calculated and compared over patients with itchy, sore, painful skin over the last week and patients who didn’t have symptoms at all over the last week. It has been shown that there was a significantly higher total score among patients with active symptoms of atopic dermatitis last week (p-value<0.001), which shows a poorer quality of sleep as compared to other subjects, as shown in Table 4.

**Table 4:** Pittsburgh Sleep Quality Index (PSQI) domains among atopic dermatitis (AD) patients with active and inactive symptoms

PSQI component	AD symptoms over the past week				p-value
	active symptoms		inactive symptoms		
	Mean	SD	Mean	SD	
Subjective sleep quality	1.3	0.2	0.8	0.1	0.023
Sleep latency	1.8	0.6	1.1	0.3	<0.001
Sleep duration	1.3	0.1	0.6	0.1	<0.001
Habitual sleep efficiency	1.9	0.4	1.2	0.5	0.036
Sleep disturbance	1.6	0.1	0.7	0.2	<0.001
Use of sleep medications	0.7	0.3	0.3	0.1	0.014
Daytime dysfunction	0.6	0.3	0.4	0.1	<0.001
Total score	7.6	2.3	2.9	1.3	<0.001

## Discussion

Atopic dermatitis is one of the most common skin conditions accompanied by significant soreness and dryness of the skin [6]. Patients with atopic dermatitis usually suffer from social embarrassment and a significant impact on their quality of life [7]. Additionally, the severity and frequency of atopic dermatitis symptoms have also been correlated to sleep disturbances; however, this correlation is still unclear in the Saudi population [8].

The present study examined the correlation of atopic dermatitis symptoms and sleep disturbances among adults living in Saudi Arabia. The study demonstrated that sleep quality was affected in at least one-third of the responders due to atopic dermatitis skin symptoms. Furthermore, 23.8% of the responders described their sleep quality as a very good quality of sleep; while 17.8% described it as very bad, 14% had a fairly bad quality of sleep. Moreover, the total score for sleep quality indicated significantly worse sleep quality in patients with active atopic dermatitis symptoms (p-value<0.001).

The impact of atopic dermatitis on sleep quality has been evaluated in different settings. Sherry et al. identified fatigue and burden accompanied by sleep among adults with atopic dermatitis in the United States [9]. Through surveying 5563 adults, respondents were asked about a history of atopic dermatitis, fatigue-related instrumental activity of daily living (IADL) impairment, and sleep disturbance. Additionally, the authors illustrated that adults with atopic dermatitis showed significantly impaired sleep and fatigue, affecting their daily life activities, and highlighted that sleep disturbances might be underdiagnosed. Similarly, our study demonstrated that 17.3% of the responders found their symptoms interfere with daily life activities. Also, 10.8% of the included patients found their symptoms affect their occupational life. 

Another study was by Kong et al., which evaluated the relationships between clinical disease severity, quality of life, and sleep quality in both children and adults with atopic dermatitis [10]. Both the Children’s Sleep Habits Questionnaire (CSHQ) and the Children’s Dermatology Life Quality Index (CDLQI) were used for children, while PSQI and DLQI were used in adult atopic dermatitis patients and demonstrated that increasing severity of AD affects sleep quality in children and significant correlation between the severity of atopic dermatitis and poor quality of life in adults, despite the insignificant correlation with sleep quality, which supports the findings of the present study.

However, the present study examined only adults, where the responders couldn’t fall asleep within half an hour (p-value=0.002), waking up at night (p-value=0.005), not breathing comfortably at night (p-value=0.018), coughing and snoring during sleep (p-value=0.011), being unable to sleep well because feeling very cold (p-value=0.037) or very hot (p-value<0.001), not being able to sleep because of pain (p-value<0.001), were all significantly correlated to the occurrence of many or a lot of skin symptoms of atopic dermatitis.

These outcomes were also congruent with Silverberg et al., which reported an association between adult atopic dermatitis and altered sleep duration, fatigue, and sleep disturbances, including daytime sleepiness and insomnia [11]. Although adults with atopic dermatitis were more likely to report fair/poor health, the presence of sleep disturbances in combination with eczema significantly increased the rates of fair/poor health.

Finally, the present study had some limitations; Face-to-face interviews were planned to take place with patients; however, due to the COVID-19 pandemic, the questionnaire was carried out online due to clinic closures. Additionally, the responses to this survey depended mainly on patients' subjective opinions, which might affect the results' reliability.

## Conclusions

Patients with atopic dermatitis significantly suffer from sleep disturbances. The extent of the affection of sleep quality is correlated to the frequency of skin symptoms. These findings can help clinicians understand the clinical manifestations and presenting symptoms of patients with atopic dermatitis. Further studies are required to explore methods to improve the quality of sleep in this particular patient population and adequate guidelines for treating or evaluating AD patients’ sleep disorders.
